# A Systematic Review and Meta-Analysis Assessing the Accuracy of Blood Biomarkers for the Diagnosis of Ischemic Stroke in Adult and Elderly Populations

**DOI:** 10.1523/ENEURO.0302-24.2024

**Published:** 2024-11-07

**Authors:** Suebsarn Ruksakulpiwat, Wendie Zhou, Lalipat Phianhasin, Chitchanok Benjasirisan, Tingyu Su, Heba M. Aldossary, Aaron Kudlowitz, Abhilash K. Challa, Jingshu Li, Kulsatree Praditukrit

**Affiliations:** ^1^Department of Medical Nursing, Faculty of Nursing, Mahidol University, Bangkok 10700, Thailand; ^2^School of Nursing, Peking University, Beijing 100191, China; ^3^The Faculty of Medicine and Health, The University of Sydney, New South Wales 2006, Australia; ^4^Department of Nursing, Prince Sultan Military College of Health Sciences, Dhahran 34313, Saudi Arabia; ^5^The College of Arts and Sciences, Case Western Reserve University, Cleveland, Ohio 44106; ^6^Rocky Vista University College of Osteopathic Medicine, Ivins, Utah 84738; ^7^Hemodialysis Center, Second Affiliated Hospital of Harbin Medical University, Harbin 150086, China; ^8^Department of Neurology, SUNY Downstate Health Sciences University, Brooklyn, New York 11203

**Keywords:** blood biomarker, diagnostic test, ischemic stroke, meta-analysis

## Abstract

This study aims to elucidate the methodology and compare the accuracy of different blood biomarkers for diagnosing ischemic stroke (IS). We reviewed 29 articles retrieved from PubMed, MEDLINE, Web of Science, and CINAHL Plus with Full Text. Among these, 23 articles involving 3,494 participants were suitable for meta-analysis. The pooled area under the curve (AUC) of all studies for meta-analysis was 0.89. The pooled sensitivity and specificity were 0.76 (0.74–0.78) and 0.84 (0.83–0.86), respectively. Blood biomarkers from noninpatient settings demonstrated better diagnostic performance than those in inpatient settings (AUC 0.91 vs 0.88). Smaller sample sizes (<100) showed better performance than larger ones (≥100; AUC 0.92 vs 0.86). Blood biomarkers from acute IS (AIS) patients showed higher diagnostic values than those from IS and other stroke types (AUC 0.91 vs 0.87). The diagnostic performance of multiple blood biomarkers was superior to that of a single biomarker (AUC 0.91 vs 0.88). The diagnostic value of blood biomarkers from Caucasians was higher than that from Asians and Africans (AUC 0.90 vs 0.89, 0.75). Blood biomarkers from those with comorbidities (AUC 0.92) showed a better diagnostic performance than those not reporting comorbidities (AUC 0.84). All the subgroups analyzed, including setting, sample size, target IS population, blood biomarker profiling, ethnicity, and comorbidities could lead to heterogeneity. Blood biomarkers have demonstrated sufficient diagnostic accuracy for diagnosing IS and hold promise for integration into routine clinical practice. However, further research is recommended to refine the optimal model for utilizing blood biomarkers in IS diagnosis.

## Significance Statement

This research shows that blood tests can accurately diagnose ischemic stroke, potentially improving the speed and accuracy of current methods like brain scans. By analyzing multiple studies, we found that combining several blood biomarkers enhances diagnostic reliability. This advancement could lead to quicker, more precise diagnoses, better patient outcomes, and more efficient stroke care. Our findings suggest a significant step forward in stroke diagnosis, highlighting the need for further research to integrate these blood tests into routine clinical practice.

## Background

Stroke is a leading cause of mortality and disability (Y. Chen et al., 2020). Previous literature stated that 5.5 million deaths and 116.4 million disability-adjusted life years) were due to stroke in 2016 (Feigin et al., 2019). According to the National Center for Chronic Disease Prevention and Health Promotion, ischemic stroke (IS) is the most prevalent stroke incident, accounting for roughly 80% of all strokes (Virani et al., 2021). IS occurs when blood flow through the artery that supplies oxygen-rich blood to the brain becomes obstructed. Currently, physicians primarily use brain imaging, such as computed tomography (CT) scan or magnetic resonance imaging (MRI) for IS diagnosis (Chalela et al., 2007; Whiteley et al., 2008). However, the limitations of these technologies must be addressed. For example, interpreting a CT scan can be challenging at the hospital as it is often normal after the onset of IS and may remain normal in patients with mild IS. Moreover, although MRI seems more sensitive in IS detection, it is not entirely accurate and may not be immediately available in acute cases (Chalela et al., 2007; Whiteley et al., 2008). Early IS diagnosis and treatment are crucial determinants of successful interventions in patients with suspected acute stroke. Even with relatively mild symptoms, patients with IS may be qualified for intravenous thrombolysis or other means of brain reperfusion if treatment can be started within a few hours of symptom onset (Johnston et al., 2007). On the other hand, delays in care after an acute IS (AIS) can cause poorer clinical outcomes and correlate with loss of healthy life years (Lacy et al., 2001). Therefore, alternative approaches that achieve acceptable sensitivity and specificity in distinguishing between stroke and mimic stroke and between stroke subtypes should be reconsidered, especially in prehospital stroke management, to provide proper treatment early. Blood biomarkers, an objective measurement of molecular characteristics, have been proposed as a tool to help in acute stroke diagnosis (Bustamante et al., 2017). However, the development of blood biomarkers for IS is facing difficulty because the release of glial and neuronal proteins was delayed due to the slow release of brain issue protein into the blood after stroke through the blood–brain barrier. Furthermore, other conditions, such as severe myocardial infarction or brain infection, which show the blood biomarkers of ischemic and infractions, could mimic stroke (Whiteley et al., 2008). Therefore, as translational medical research has provided much effort in discovering the blood biomarkers for the diagnosis of IS, we believe that our systematic review and meta-analysis aiming to evaluate the diagnostic value of different blood biomarkers for the diagnosis of IS are needed. Moreover, this would help to improve the design and the report of future studies of blood biomarkers for the diagnosis of IS.

## Materials and Methods

### Search strategy

The Preferred Reporting Items for Systematic Reviews and Meta-Analyses (PRISMA; Moher et al., 2009) were applied in this systematic review to present the literature's flow diagram of the identification, screening, exclusion, and inclusion. Four electronic databases, PubMed, MEDLINE, Web of Science, and CINAHL Plus with Full Text, were systematically searched to identify preliminary studies published between 2017 and 2023, reporting blood biomarkers for the diagnosis of IS. This time frame was selected to ensure the inclusion of the most recent advancements and findings, reflecting current technological, methodological, and diagnostic standards. We combined the search terms using Boolean phrases (http://dx.doi.org/10.13140/RG.2.2.21274.45768). In addition, reference lists of the included studies were manually searched to obtain relevant studies. All references identified were stored in EndNote.

### Selection of studies

Two of this systematic review's authors independently screened titles and abstracts of eligible studies. Subsequently, the full text was also assessed to decide whether or not it was relevant. A third author was required to resolve disagreements when discrepancies occurred. Inclusion criteria were implemented to guarantee that only studies considered relevant to our objective were included. Similarly, exclusion criteria were used to eliminate literature not affiliated with the review (Table 1).

**Table 1.** Inclusion and exclusion criteria

Inclusion criteria
Human participants of age ≥18 years old (both male and female) Original studies primarily aimed to investigate blood biomarkers for the diagnosis of IS, including prospective comparative studies, cohort, case control, case series or case reports (≥10 patients), and diagnostic randomized controlled trials. Case series and case reports were excluded from the meta-analysis and included only in the review Original studies reporting the following result: TP, FP, TN, FN, sensitivity, specificity, PLR, NLR, the AUC, or DOR, or data suffice to construct two-by-two contingency tables to compute the diagnostic accuracy Included participants diagnosed with IS, including IS (thrombotic strokes or embolic strokes) or TIA All types of settings are acceptable, including inpatient, outpatient, community, or home Described in the English language Full-text availability
Exclusion criteria
The study did not include the population of interest or concerned animal subjects Conference proceedings, abstracts, review articles, theoretical papers, pilot studies, protocols, dissertations, letters to the editor, opinions (viewpoint), statement papers, government documents, or working papers

### Quality assessment

In this study, the Quality Assessment and Diagnostic Accuracy Studies-2 (QUADAS-2) tool was adopted for the quality appraisal of included studies (Whiting et al., 2011), including (1) patient selection, (2) index test, (3) reference standard, and (4) flow and timing. Each domain is assessed regarding the risk of bias, and the first three are also assessed in terms of concerns regarding applicability. Two researchers assessed the quality of the eligible studies independently. A third researcher was required when there was any discrepancy (http://dx.doi.org/10.13140/RG.2.2.27985.34404).

### Data extraction

The summary data (Table 2) included the following data for each study: reference, published year, country, study design, the study set up, study setting, sample size, target IS population, comorbidities, age, sex, prevalence of IS, all included blood biomarkers, blood biomarkers with optimal performance, true positive (TP), false negative (FN), true negative (TN), false positive (FP), sensitivity, specificity, and area under the curve (AUC) of blood biomarker with optimal performance, respectively. Moreover, Table 3 shows the summarized results of the meta-analysis, which included the following: subgroup, number of included studies, total sample size, AUC, sensitivity, specificity, positive likelihood ratio (PLR), negative likelihood ratio (NLR), and diagnostic odds ratio (DOR).

**Table 2.** The summary data

- Reference - Published Year - Country	Study design	- Study setup - Setting	- Sample size (total) (IS/C)) (*n*)^a^ - Target IS population - Comorbidities - Age (median or mean) (range) (in a year) - Male/female (*n*/*n*)	Prevalence of IS (%)	All included blood biomarkers	Blood biomarker with optimal performance	Blood biomarker with optimal performance						
							TP	FP	TN	FN	Sen (95% CI)	Spe (95% CI)	AUC (95% CI)
- (Fan et al., 2018) - 2018 - China	Prospective study	- Single-center - Inpatient	- 389 (133/144) - Acute lacunar infarction - Comorbidities: Hypertension, diabetes mellitus, hyperlipidemia, CAD, and atrial fibrillation - Acute lacunar infarction 69 (59.5–75.5), control 66.5 (59.25–71) - 227/112	34.19%	- Homocysteine (Hcy) - Fibrinogen	Hcy	128	0	192	69	0.65 (0.58–0.72)	1.00 (0.98–1.00)	0.88 (0.84–0.92)
- (Q. X. Qin et al., 2018) - 2018 - China	Retrospective study	- Multicenter - Inpatient	- 228 (114/114) - AIS with active colorectal cancer (CRCIS) - Comorbidities: Colorectal cancer - CRCIS 65.33 ± 12.45, healthy control (HC) 63.67 ± 9.18 - 156/72	50.00%	- D-dimer - Neutrophil count (NC) - Carcinoembryonic antigen (CEA) - Cancer antigen 125 (CA125)	D-dimer + CEA + NC	98	23	91	16	0.86 (0.78–0.92)	0.80 (0.71–0.87)	0.89 (0.85–0.93)
- (Fang et al., 2018) - 2018 - China	Retrospective, case-control study	- Single-center - Inpatient	- 462 (262/200) - AIS - Comorbidities: Hypertension, diabetes, hyperlipidemia - AIS 62.5 ± 11.5, HC 61.8 ± 10.8 - 261/201	56.71%	- S100 calcium-binding protein B (S100B) - C-reactive protein (CRP) - IL-6 - Plasminogen activator inhibitor-1 (PAI-1) - Matrix metallopeptidase 9 (MMP-9) - P-selectin - Intercellular adhesion molecule 1 (ICAM-1) - Tumor necrosis factor α (TNF-α) - Low-density lipoprotein cholesterol - IL-10 - Nitric oxide (NO) - Glial fibrillary acidic protein	CRP	NA	NA	NA	NA	NA	NA	0.99 (0.98–1.00)
						IL-6	NA	NA	NA	NA	NA	NA	0.96 (0.94–0.98)
						PAI-1	NA	NA	NA	NA	NA	NA	0.99 (0.98–1.00)
						P-selectin	NA	NA	NA	NA	NA	NA	0.91 (0.88–0.94)
						TNF-α	NA	NA	NA	NA	NA	NA	0.99 (0.98–1.00)
- (Mohamed et al., 2019) - 2019 - Egypt	A prospective, case-control study	- Single-center - Inpatient	- 96 (48/48) - CES - Comorbidities: N/A - NA - NA	50.00%	- Brain natriuretic peptide (BNP) - D-dimer - Creatine–kinase-MB (CK-MB) - CRP - Globulin/albumin ratio	BNP	36	18	30	12	0.75 (0.60–0.86)	0.63 (0.47–0.76)	0.80 (0.71–0.89)
						D-dimer	44	16	32	4	0.91 (0.79–0.97)	0.66 (0.51–0.79)	0.79 (0.70–0.88)
						CK-MB	41	12	36	7	0.85 (0.71–0.93)	0.75 (0.60–0.86)	0.91 (0.85–0.97)
- (Zuo et al., 2020) - 2020 - China	A prospective, cohort study	- Dual-center - Inpatient	- 300 (200/100) - AIS - Comorbidities: N/A - AIS 72 (61–82), HC 64 (59–71) - 188/112	66.67%	- circFUNDC1 - circPDS5B - circCDC14A - Combination of the above three circRNAs	Combination of three circRNAs (circFUNDC1, circPDS5B, circCDC14A)	144	9	91	56	0.72 (0.65–0.78)	0.91 (0.83–0.96)	0.88 (0.84–0.91)
- (O'Connell et al., 2017) - 2017 - USA	Prospective study	- Single-center - Inpatient	- 63 (43/20) - AIS - Comorbidities: Hypertension, dyslipidemia, diabetes, atrial fibrillation - AIS 72.5 ± 15.5, mimic stroke 58.0 ± 17.0 - 29/31	68.25%	Peripheral blood cell-free DNA (cfDNA)	cfDNA	37	5	15	6	0.86 (0.72–0.95)	0.75 (0.51–0.91)	0.90 (0.72–0.95)
- (Nezu et al., 2022) - 2022 - Japan	Prospective study	- Multicenter - Inpatient	- 120 (47/73) - CAS - Comorbidities: Hypertension, diabetes mellitus, dyslipidemia, chronic kidney disease, atrial fibrillation, cancer - AIS patients with active cancer and CAS 72.4 ± 11.5, AIS patients with active cancer 76.3 ± 10.2 - 74/46	39.17%	- Carbohydrate antigen (CA) 125 - CEA - CA 19–9	CA 125	35	16	57	12	0.75 (0.59–0.86)	0.78 (0.67–0.87)	0.81 (0.72–0.88)
- (Comertpay et al., 2020) - 2020 - Turkey	A prospective, case-control study	- Single-center - Inpatient	- 72 (36/36) - AIS - Comorbidities: N/A - AIS 66.06 ± 14.59 (23–94), HC 66.1 ± 14.4 (25–94) - 46/26	50.00%	Soluble tumor necrosis factor-like weak inducer of apoptosis (sTWEAK)	sTWEAK	28	3	33	8	0.78 (0.60–0.89)	0.92 (0.76–0.98)	0.84 (0.74–0.94)
- (C. Tian et al., 2022) - 2022 - China	A prospective, case-control study	- Single-center - Inpatient	- 78 (39/39) - AIS - Comorbidities: Diabetes mellitus, hypertension, hyperlipidemia, coronary heart disease - AIS 74, control 76.62 ± 10.34 - 36/42	50.00%	- nc-KRTCAP3-2:1 - Inc-OSBPL10 - nc-OSBPL10-2:1 - NR_120420 - Inc-AP002414.1.1 - nc-AP002414.1.1-5:8 - nc-GCH1-2:3 - NR_003529. - Inc-DENR - nc-DENR-2:3 - nc-CMPK2-5:39	NR_120420 (total anterior circulation infarction subgroup)	34	6	33	5	0.86 (0.69–0.94)	0.85 (0.69–0.94)	0.86 (0.73–0.99)
- (P. F. Liu et al., 2017) - 2017 - China	A prospective, case-control study	- Single-center - Inpatient	- 49 (26/23) - AIS - Comorbidities: hypertension, hyperlipidemia - AIS 59.08 ± 8.51, control 56.96 ± 2.70 - 33/16	53.06%	- Ornithine - Proline - Acetylcarnitine - Threonine - PC (1:0/16:0) - LysoPC (16:0) - LysoPE (20:4) - Carnitine - Lysine - Aspartic acid - LysoPE (18:2) - Betaine - Isoleucine - Serine PC (5:0/5:0)	Combination of five biomarkers (serine, isoleucine, betaine, PC (5:0/5:0), LysoPE (18:2))	NA	NA	NA	NA	NA	NA	0.97 (0.92–1.02)
- (Cheng et al., 2018) - 2018 - China	A prospective, case-control study	- Single-center - Inpatient	- 119 (77/42) - AIS - Comorbidities: Hypertension, diabetes mellitus - AIS 61 ± 10.4, control 59 ± 4.7 - 35–79 (61) - 83/36	64.71%	- miR-148b-3p - miR-151b - miR-27b-3p	Combination of miR-148b-3p and miR-27b-3p	52	3	39	25	0.67 (0.46–0.67)	0.93 (0.70–0.97)	0.81 (0.70–0.92)
- (H. Tian et al., 2021) - 2021 - China	A prospective, case-control study	- Single-center - Inpatient	- 140 (76/64) - AIS - Comorbidities: Hypertension, diabetes, hyperlipidemia - AIS 57.0 ± 6.4, control 56.4 ± 6.2 - 87/53	54.29%	- miR-210 - miR-137 - miR-153	miR-210	46	14	62	18	0.72 (0.59–0.82)	0.82 (0.71–0.89)	0.82 (0.76–0.91)
						miR-137	45	11	65	19	0.70 (0.57–0.81)	0.86 (0.75–0.92)	0.84 (0.78–0.91)
						miR-153	52	20	56	12	0.81 (0.69–0.90)	0.74 (0.62–0.83)	0.84 (0.78–0.91)
- (Wang et al., 2017) - 2017 - China	A prospective, case-control study	- Single-center - Inpatient	- 69 (40/29) - AIS - Comorbidities: Hypertension, diabetes mellitus, atrial fibrillation - AIS 65.7, control 61.0 - 38/31	57.97%	- Alanine - Citrate - Glycine - Leucine - Isoleucine - Serine - Tyrosine - Methionine - Tryptophan - Erythronic acid - Urea - Purine - Proline - Hypoxanthine	Tyrosine, lactate, and tryptophan panel	NA	NA	NA	NA	NA	NA	NA
- (D. Lu et al., 2020) - 2020 - China	Phase 1: Experimental design Phase 2: Bioinformatics analysis, case-control study	- Multicenter - Inpatient	- 16 (8/8) - AIS - Comorbidities: N/A - AIS 55.4 ± 12.3, control 54.11 ± 10.1 - 14/2	50.00%	140,732 out of 140,790 human circRNAs	- circPHKA2 - circBBS2	NA	NA	NA	NA	NA	NA	NA
- (Augello et al., 2018) - 2018 - USA	A prospective, case-control study	- Single-center - Inpatient and outpatient	- 50 (24/26) - AIS - Comorbidities: Hypertension, CAD, CAD, diabetes mellitus, atrial fibrillation - AIS 58 (51–74), control 61 (51–76) - 31/19	48.00%	- Gp130/sIL-6Rb - IFN-β - IL-28A/IFN-λ2 - MMP-2 - Osteopontin (OPN) - sTNF-R1 - sTNF-R2 - TSL	IL-28A/IFN-λ2, MMP-2, sTNF-R2, and TSLP panel	21	1	25	3	0.88 (0.67–0.97)	0.96 (0.78–1.00)	0.96 (0.89–1.02)
- (Guo et al., 2018) - 2018 - China	A prospective, case-control study	- Single-center - Inpatient	- 20 (10/10) - AIS - Comorbidities: N/A - AIS 63.2 ± 10.7, control 69.3 ± 5.9 - 14/6	50.00%	560 upregulated and 690 downregulated differentially expressed lncRNAs	Combination of lncRNAs (lncRNA-ENST00000568297, lncRNA-ENST00000568243, and lncRNA-NR_046084)	8	2	8	2	0.83 (0.44–0.96)	0.80 (0.44–0.96)	0.84 (0.66–1.02)
- (Huang et al., 2021) - 2021 - China	A prospective, cohort study	- Single-center - Inpatient	- 305 (155/150) - AIS - Comorbidities: Hypertension - AIS 56.0 (median), control 55.0 (median) - AIS 84/71, control 76/74	50.82%	- Serum Interleukin-34 (IL-34) - Glycosylated hemoglobin (HbA1c)	IL-34	106	5	145	49	0.68 (0.60–0.75)	0.97 (0.92–0.98)	0.87 (0.83–0.91)
- (L. J. Zhang et al., 2020) - 2020 - China	Prospective study	- NA - NA	- 78 (48/30) - Fist-ever IS (FS) and recurrent IS (RS) - Comorbidities: High blood pressure, coronary heart disease, valvular disease of the heart, heart failure, atrial fibrillation - FS 64.2 ± 8.9, RS 75.8 ± 9.4, HC 65.5 ± 8.2 - FS 21/17, RS 7/3, HC 17/13	61.54%	- Model 1: fatty acid metabolite levels including DHA, 8-iso-PGF3α, arachidonic acid, 8-iso-15-keto-PGF2a, 13-HODE, and 14,15-DHET - Model 2: clinical biochemical parameter levels, including HDL, blood glucose, and TG - Model 3: both fatty acid metabolites and clinical biochemical parameters: arachidonic acid, DHA, 13-HODE, 8-iso-15-keto-PGF2a, and HDL	Model 3: both fatty acid metabolites and clinical biochemical parameters: arachidonic acid, DHA, 13-HODE, 8-iso-15-keto-PGF2a, and HDL	48	2	28	0	1.00 (0.91–1.00)	0.93 (0.77–0.99)	0.99 (0.98–1.00)
- (Rahmati et al., 2021) - No. 22 - 2021 - Iran	Prospective study	- Single-center - Inpatient	- 104 (52/52) - IS - Comorbidities: N/A - NA - NA	50.00%	- micronRNA-210 (miR-210) - Hypoxia inducible factor-1α (HF-1α)	HF-1α	34	20	32	18	0.65 (0.50–0.78)	0.62 (0.47–0.75)	0.73 (0.64–0.82)
- (Zhao et al., 2021) - 2021 - China	Prospective study	- Single-center - Inpatient	- 100 (50/50) - IS - Comorbidities: Hypertension, diabetes mellitus - IS 66.76 (median), control 66.74 (median) - IS 31/19, control 30/20	50.00%	- microRNA-148a (has-miR-148a) - microRNA-342-3p (has-miR-342-3p) - microRNA-19a (has-miR-19a) - microRNA-320d (has-miR320d) - has-miR-148a + has-miR-342-3p - has-miR148a + has-miR-342-3p + has-miR-19a - has-miR148a + has-miR-342-3p + has-miR-19a + has-miR-320d	has-miR148a + has-miR-342-3p + has-miR-19a + has-miR-320d	47	9	41	3	0.94 (0.83–0.98)	0.82 (0.68–0.91)	0.89 (0.77–1.00)
- (Park et al., 2018) - 2017 - USA	A prospective, case-control study	- Multicenter - Inpatient and outpatient	- 305 (172/133) - AIS - Comorbidities: Hypertension, diabetes mellitus, hyperlipidaemia, CAD, atrial fibrillation - AIS 68.8 ± 14.7, control 71.0 ± 10.5 - 147/158	56.39%	- Glycogen phosphorylase isoenzyme BB (GPBB)	Glycogen phosphorylase isoenzyme BB (GPBB)	160	9	124	12	0.93 (0.88–0.96)	0.93 (0.87–0.97)	0.96 (0.94–0.98)
- (H. T. Zhang et al., 2020) - 2020 - China	Case-control study	- Single-center - Inpatient	- 163 (93/70) - IS - Comorbidities: Hypertension, diabetes mellitus - IS 67.5 ± 11.2, control 66.2 ± 10.8 - 96/67	57.06%	- Endothelial microvesicles (EMVs) - EMVs carrying miRNA-155	EMVs + EMVs-miR-155	70	8	85	23	0.75 (0.65–0.83)	0.91 (0.83–0.96)	0.89 (0.84–0.94)
- (Sun et al., 2017) - 2017 - China	Case-control study	- Single-center - Inpatient and outpatient	- 60 (30/30) - IS - Comorbidities: N/A - IS 59.77 ± 12.41, control 57.83 ± 11.08 - 34/26	50.00%	12 Metabolites - Uric acid - LysoPE (0:0/18:3) - LysoPC (18:2) - Bilirubin - Sphinganine - Linoelaidyl carnitine - LysoPC (16:0) - Adrenoyl ethanolamide - PE (15:0/22:1) - PS (14:1/22:6) - PC (14:0/20:4) - PC (16:0/22:6)	Uric acid	22	7	23	8	0.73 (0.54–0.87)	0.77 (0.57–0.89)	0.78 (0.66–0.90)
						Sphinganine	21	5	25	9	0.70 (0.50–0.85)	0.83 (0.65–0.94)	0.75 (0.63–0.88)
						Adrenoyl ethanolamide	23	7	23	7	0.77 (0.57–0.89)	0.77 (0.57–0.89)	0.81 (0.70–0.92)
- (C. Qin et al., 2019) - 2019 - China	- A retrospective, case-control study	- Single-center - Inpatient	- 66 (33/33) - AIS due to large-vessel occlusion (LVO) - Comorbidities: Hypertension, diabetes mellitus, coronary heart disease, hypercholesterolemia - AIS due to LVO 54 (46–59), HC 55 (45–62) - 41/25	50.00%	- IGF2 - LYVE1 - PPBP, THBS1	The 4-protein panel (IGF2, LYVE1, PPBP, THBS1)	NA	NA	NA	NA	NA	NA	0.95 (0.90–1.01)
- (Lee et al., 2020) - 2020 - South Korea	A prospective, case-control study	- Single-center - Inpatient	- 61 (30/31) - AIS - Comorbidities: Cardiovascular disease, hypertension, diabetes, atrial fibrillation - AIS 59.9 ± 10.9, HC 56.12 ± 5.6 - 30/31	49.18%	- Prothrombin (F2) - Plasminogen (PLG) - Fibrinogen alpha chain (FGA) - Histidine-rich glycoprotein (HRG)	F2	26	4	27	4	0.87 (0.68–0.96)	0.87 (0.69–0.96)	0.92 (0.84–0.99)
						PLG	29	1	30	1	0.97 (0.81–1.00)	0.97 (0.82–1.00)	0.98 (0.94–1.02)
						FGA	27	2	29	3	0.90 (0.72–0.97)	0.93 (0.77–0.99)	0.92 (0.85–0.99)
						HRG	29	1	30	1	0.97 (0.81–1.00)	0.97 (0.815–1.00)	0.98 (0.944–1.02)
- (Tiedt et al., 2017) - 2017 - Germany	Case-control study	- Single-center - Inpatient	- 300 (200/100) - IS - Comorbidities: Hypertension, hypercholesterolemia, diabetes mellitus - IS 74.1 ± 13.4, HC 65.6 ± 13.4 - 148/152	66.67%	- 33 microRNAs (miRNAs)	A set of miR-125a-5p, miR-125b-5p and miR-143-3p	171	24	76	29	0.86 (0.80–0.90)	0.76 (0.66–0.84)	0.90 (0.87–0.93)
- (L. S. Dolmans et al., 2019) - 2019 - the Netherlands	Cross-sectional study	- NA - Outpatient and home	- 206 (126/80) - TIA - Comorbidities: Hypertension, diabetes mellitus, hyperlipidemia, cerebrovascular disease, renal insufficiency, migraine, epilepsy - TIA 71.4 ± 12.0, control 62.0 ± 14.2 - 122/84	61.17%	- NR2 - NR2Ab - B-FABP - H-FABP - NDKA - UFD1 - PARK7	H-FABP	44	28	52	82	0.35 (0.27–0.44)	0.65 (0.53–0.75)	NA
- (J. Liu et al., 2020) - 2020 - China	Prospective study	- Single-center - Inpatient	- 137 (72/65) - AIS - Comorbidities: Hypertension, diabetes mellitus, dyslipidemia, cervical artery plaques - AIS 58.7 ± 9.7, control 56.6 ± 8.5 - 81/56	52.55%	- Sphingosine 1-phosphate (S1P) - High-density lipoprotein cholesterol (HDL-C)	S1P	44	25	40	28	0.61 (0.49–0.72)	0.62 (0.49–0.73)	0.62 (0.53–0.72)
						S1P within 24 h of symptom onset	49	8	57	23	0.68 (0.56–0.78)	0.87 (0.77–0.94)	0.83 (0.74–0.92)
- (Z. Z. Chen et al., 2018) - 2018 - China	Prospective study	- NA - Inpatient	- 230 (128/102) - AIS - Comorbidities: Hypertension, diabetes, coronary disease, dyslipidemia - AIS 68.42 ± 17.26, control 65.36 ± 16.32 - 109/19	55.65%	- Plasma hs-CRP - Serum miR-146b, miR-21, miR-145, miR-29b, miR-221 - Interleukin (IL-6)	Combination of hs-CRP, IL-6 and miR-146b	NA	NA	NA	NA	NA	NA	0.87 (0.80–0.93)

IS, ischemic stroke group; C, control group; TP, true positive; FP, false positive; TN, true negative; FN, false negative; Sen, sensitivity; Spe, specificity; AUC, area under the curve; N/A, not applicable.

a The one generated the final diagnostic value.

**Table 3.** Summarized results of the meta-analysis

Subgroup	Number of included studies	Total sample size	AUC	Sen (95% CI)	Spe (95% CI)	PLR (95% CI)	NLR (95% CI)	DOR (95% CI)
All combined	23	3,494	0.89	0.76 (0.74–0.78)	0.84 (0.83–0.86)	4.91 (3.69–6.53)	0.26 (0.20–0.32)	23.14 (14.15–37.84)
Setting								
Inpatient	18	2,795	0.88	0.76 (0.74–0.78)	0.84 (0.83–0.86)	4.85 (3.64–6.45)	0.28 (0.23–0.33)	21.66 (14.15–33.16)
Others^a^	4	621	0.91	0.71 (0.66–0.75)	0.83 (0.78–0.87)	4.40 (1.54–12.60)	0.27 (0.09–0.83)	16.95 (2.41–119.25)
Sample size								
<100	9	578	0.92	0.86 (0.82–0.88)	0.82 (0.79–0.86)	4.95 (3.44–7.12)	0.20 (0.14–0.27)	30.87 (16.31–58.42)
≥100	14	2,916	0.86	0.73 (0.71–0.75)	0.85 (0.83–0.87)	4.73 (3.11–7.19)	0.31 (0.23–0.41)	17.57 (8.84–34.91)
Target IS population								
IS	6	805	0.87	0.82 (0.78–0.85)	0.80 (0.76–0.84)	4.07 (2.65–6.27)	0.26 (0.17–0.40)	17.74 (7.85–40.10)
AIS	12	1,650	0.91	0.77 (0.75–0.80)	0.88 (0.85–0.90)	6.78 (4.40–10.44)	0.24 (0.19–0.31)	32.72 (17.38–61.60)
Others^b^	5	1,039	0.84	0.68 (0.64–0.72)	0.81 (0.78–0.84)	3.00 (1.66–5.42)	0.31 (0.17–0.56)	12.91 (3.69–45.16)
Blood biomarker profiling								
Single	14	2,136	0.88	0.73 (0.71–0.75)	0.84 (0.82–0.86)	4.52 (3.15–6.47)	0.27 (0.21–0.36)	19.74 (10.56–36.89)
Multiple	9	1,358	0.91	0.81 (0.78–0.83)	0.86 (0.83–0.88)	5.89 (4.23–8.20)	0.22 (0.16–0.30)	30.20 (19.44–46.91)
Ethnicity								
Asian	17	2,474	0.89	0.75 (0.73–0.77)	0.86 (0.85–0.88)	5.55 (4.00–7.68)	0.29 (0.25–0.35)	23.92 (14.81–38.64)
Caucasian	5	924	0.90	0.77 (0.73–0.80)	0.81 (0.77–0.85)	4.53 (1.56–13.14)	0.21 (0.06–0.77)	23.52 (2.93–188.62)
African	1	96	0.75	0.84 (0.77–0.90)	0.68 (0.60–0.76)	2.59 (1.92–3.50)	0.23 (0.12–0.46)	11.76 (4.52–30.56)
Comorbidities								
With comorbidities	16	2,537	0.92	0.77 (0.75–0.79)	0.85 (0.83–0.87)	5.56 (3.82–8.09)	0.22 (0.16–0.31)	31.55 (16.00–62.20)
N/A	7	957	0.84	0.74 (0.70–0.77)	0.82 (0.79–0.85)	3.99 (2.55–6.25)	0.32 (0.27–0.39)	14.21 (7.71–26.19)

Sen, sensitivity; Spe, specificity; PLR, positive likelihood ratio; NLR, negative likelihood ratio; AUC, area under the curve; DOR, diagnostic odds ratio; IS, ischemic stroke; AIS, acute ischemic stroke; N/A, not applicable.

a Inpatient and outpatient, outpatient and home.

b Acute lacunar infarction, CRCIS, CES, CAS, TIA.

### Statistical analysis

The statistical analysis was performed using the Meta-DiSc 1.4 software (Ramón y Cajal Hospital), ReviewManager (RevMan 5.3, The Cochrane Collaboration, Software Update), and STATA 12.0 (STATA). TP, FP, TN, and FN were back-calculated with the sample size, sensitivity, and specificity. Heterogeneity was measured using the *I*^2^ test (*I*^2 ^> 50%, significant heterogeneity) and Cochrane's *Q* test (*p* < 0.05, heterogeneity). The random-effect model was used if *I*^2 ^> 50% or *p* < 0.05 (Higgins and Thompson, 2002); otherwise, the fixed-effect model was adopted. The threshold effect was assessed by the Spearman correlation coefficient and *p* value using Meta-DiSc (*p* < 0.05, threshold effect; X. Lu et al., 2019). The pooled AUC of the summary receiver operating characteristic curve (SROC) was calculated by Meta-DiSc (0.5 ≤ AUC < 0.7, low; 0.7 ≤ AUC < 0.8, acceptable; 0.8 ≤ AUC < 0.9, moderate; 0.9 ≤ AUC = 1, high; Swets, 1988), along with the pooled sensitivity, specificity, PLR, NLR, and DOR (>1, useful for diagnosing). We added 1/2 to all the cells of all studies to deal with empty cells (Hasselblad and Hedges, 1995). The quality appraisal result based on QUADAS-2 was generated by RevMan 5.3.

Subgroups were analyzed by Meta-DiSc for potential sources of heterogeneity based on (1) setting, (2) sample size, (3) target IS population, (4) blood biomarker profiling, (5) ethnicity, and (6) comorbidities. Meta-regression analysis was conducted by STATA for heterogeneity (*p* < 0.05, significant contribution to heterogeneity). Deeks’ funnel plot asymmetry test was applied to assess potential publication bias by STATA (*p* < 0.1, significant asymmetry and a publication bias; Deeks et al., 2005). The Fagan nomogram was created by STATA to evaluate the clinical utility of blood biomarkers for the diagnosis of IS.

## Results

### Searched results

**Figure 1.**
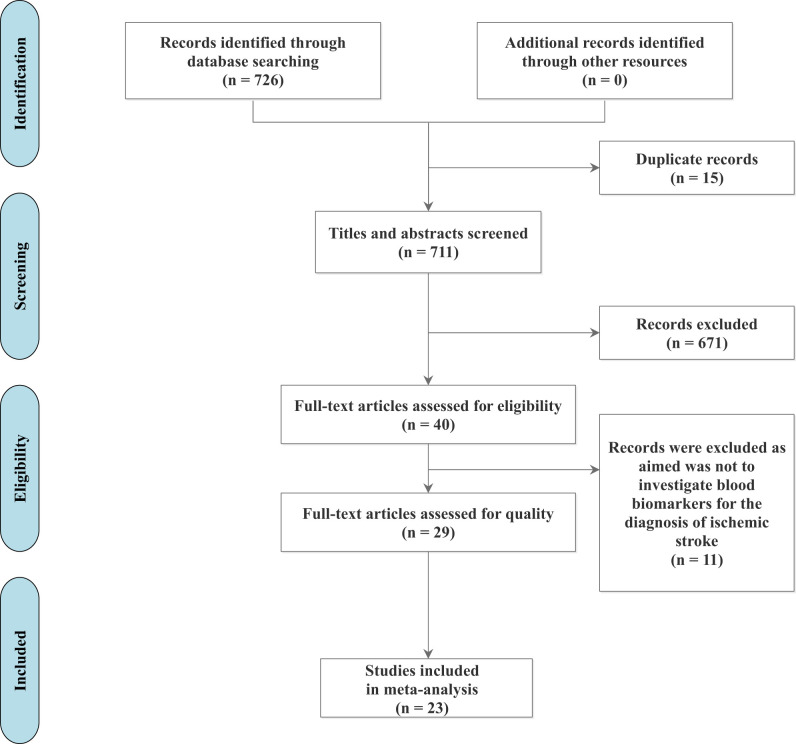
PRISMA flow chart.

An initial search of the literature generated 726 articles, with no additional records identified through other resources. Among these, 15 duplicates were identified and eliminated. After deduplication, the researchers prepared 711 references for screening, of which 671 articles were excluded during the title and abstract screening phase following the inclusion and exclusion criteria (Table 1). This process left 40 articles eligible for full-text screening. During this phase, 11 articles were excluded for reasons such as the primary aim was not to investigate blood biomarkers for the diagnosis of IS (e.g., studies focusing on predicting stroke outcomes rather than diagnosing IS, studies combining blood biomarkers with other variables like smoking history and hypertension to diagnose IS, etc.). A total of 29 articles were included in this systematic review for final screening and quality appraisal, and 23 studies were further included in the meta-analysis. PRISMA was utilized to outline the retrieval process (Fig. 1).

### Study characteristics of included studies

Table 4 shows that all included studies were published between 2017 and 2022 and the majority of them were published in 2018 (*n* = 8 studies; 27.59%), 2020 (*n* = 7 studies; 24.14%), and 2017 (*n* = 5 studies; 17.24%). Moreover, more than half of the included studies were conducted in China (*n* = 19 studies; 65.52%). The study designs were prospective study (*n* = 21 studies; 46.67%), case-control study (*n* = 17 studies; 37.78%), retrospective study (*n* = 3 studies; 6.67%), cohort study (*n* = 2 studies; 4.44%), and cross-sectional and experimental study with *n* = 1 (2.22%) each. For the study setup, 72.41% of the included studies were single-center, and 17.24% were multicenter. More than 80% (*n* = 27 studies) of included studies were conducted in inpatient departments. Other settings, such as outpatient departments and home settings, were <10%. The sample size ranged from >50 to 100 (*n* = 10 studies; 34.48%), >100 to 200 (*n* = 6 studies; 20.69%), and >200 to 300 (*n* = 5 studies; 17.24%) that are among the most popular reported. The dominant of our target population was patients with AIS (*n* = 20 studies; 68.97%) and IS (*n* = 6 studies; 20.69%). The age of participants was reported differently across studies, ranging from 45 to 59 years (10 studies, 27.78%), 60–70 (16 studies, 44.44%), and >70 (eight studies, 22.22%). Finally, 72.41% (*n* = 21 studies) of included studies reported >40–60% prevalence of IS.

**Table 4.** The characteristics of the included studies

Characteristics	Number of included studies (*n*)^a^	Percentage (%)
Publication year		
2017	5	17.24
2018	8	27.59
2019	3	10.34
2020	7	24.14
2021	4	13.79
2022	2	6.90
Country		
China	19	65.52
USA	3	10.34
Egypt	1	3.45
Japan	1	3.45
Turkey	1	3.45
Iran	1	3.45
South Korea	1	3.45
Germany	1	3.45
Netherlands	1	3.45
Study design		
Prospective study	21	46.67
Case-control study	17	37.78
Retrospective study	3	6.67
Cohort study	2	4.44
Cross-sectional study	1	2.22
Experimental study	1	2.22
Study setup		
Single center	21	72.41
Multicenter	5	17.24
N/A	3	10.34
Setting		
Inpatient department	27	84.38
Outpatient department	3	9.38
Home	1	3.13
N/A	1	3.13
Total sample size (*n*)		
1–50	4	13.79
>50–100	10	34.48
>100–200	6	20.69
>200–300	5	17.24
>300–400	3	10.34
>400	1	3.45
Target IS population		
AIS	20	68.97
IS	6	20.69
Acute lacunar infraction	1	3.45
Cardio embolic stroke	1	3.45
TIA	1	3.45
Age of participants (mean/median in years)		
45–59	10	27.78
60–70	16	44.44
>70	8	22.22
NA	2	5.56
Prevalence of IS (%)		
>20–40	2	6.90
>40–60	21	72.41
>60–80	6	20.69

N/A, not applicable.

a One study may have ≥ 1 characteristic.

### The quality appraisal of included studies

**Figure 2.**
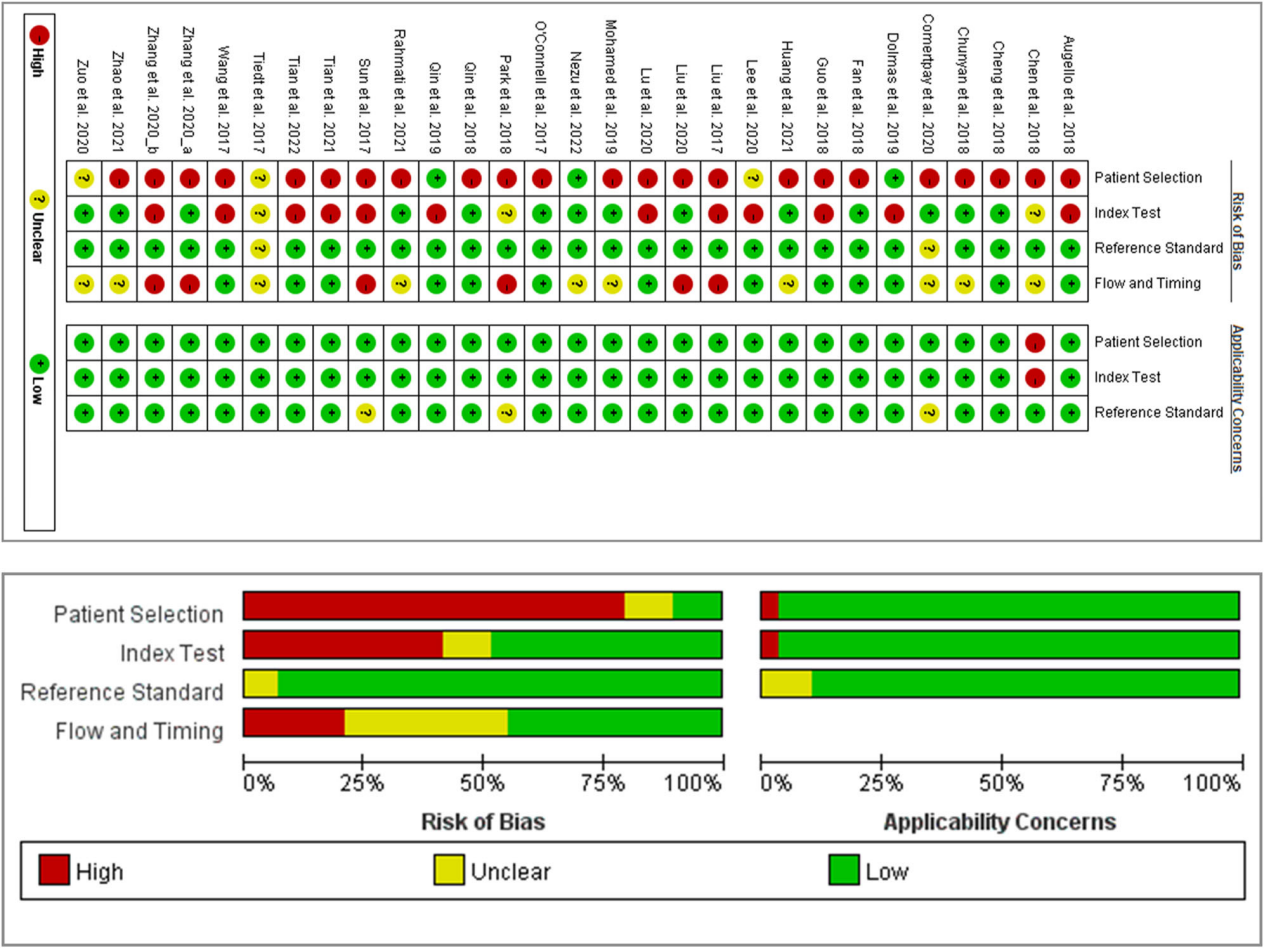
Risk of bias summary using the QUADAS-2. Zhang et al. 2020_a refers to L. J. Zhang et al., 2020, Zhang et al. 2020_b refer to H. T. Zhang et al., 2020.

The QUADAS-2 tool was adopted for the quality appraisal of included studies (http://dx.doi.org/10.13140/RG.2.2.27985.34404). Our study found that risks of bias concerning patient selection were high for most studies (>75%). As for the index test, the proportion of those with a high risk of bias was similar to those with a low risk of bias. Almost all studies showed low risks of bias for the reference standard. Regarding flow and timing, studies of low risks of bias and those of unclear risks of bias shared almost the same proportions. Moreover, applicability concerns were low in most studies for patient selection, index test, and flow and timing. Details of the risk of bias and applicability concerns in each study are shown in Figure 2.

### Diagnostic performance of blood biomarkers

**Figure 3.**
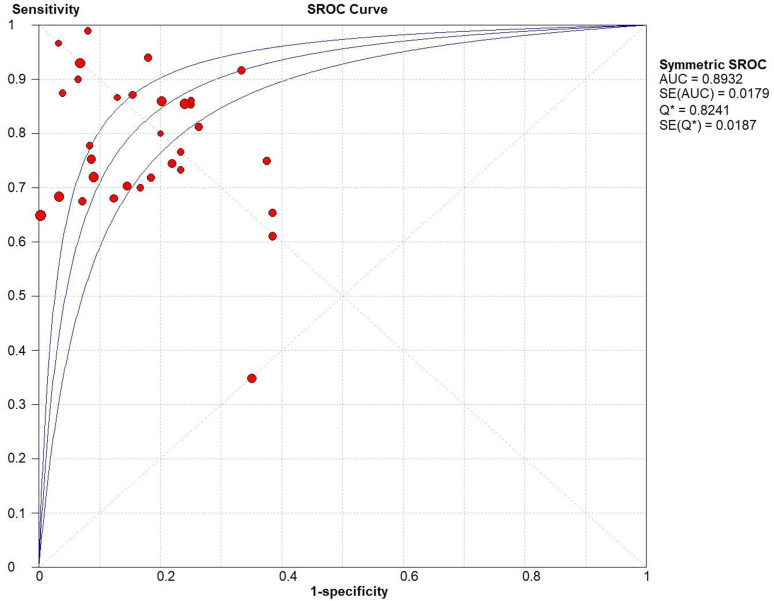
SROC for all studies. AUC, 0.89. SROC, summary receiver operating characteristic; AUC, area under the curve.

**Figure 4.**
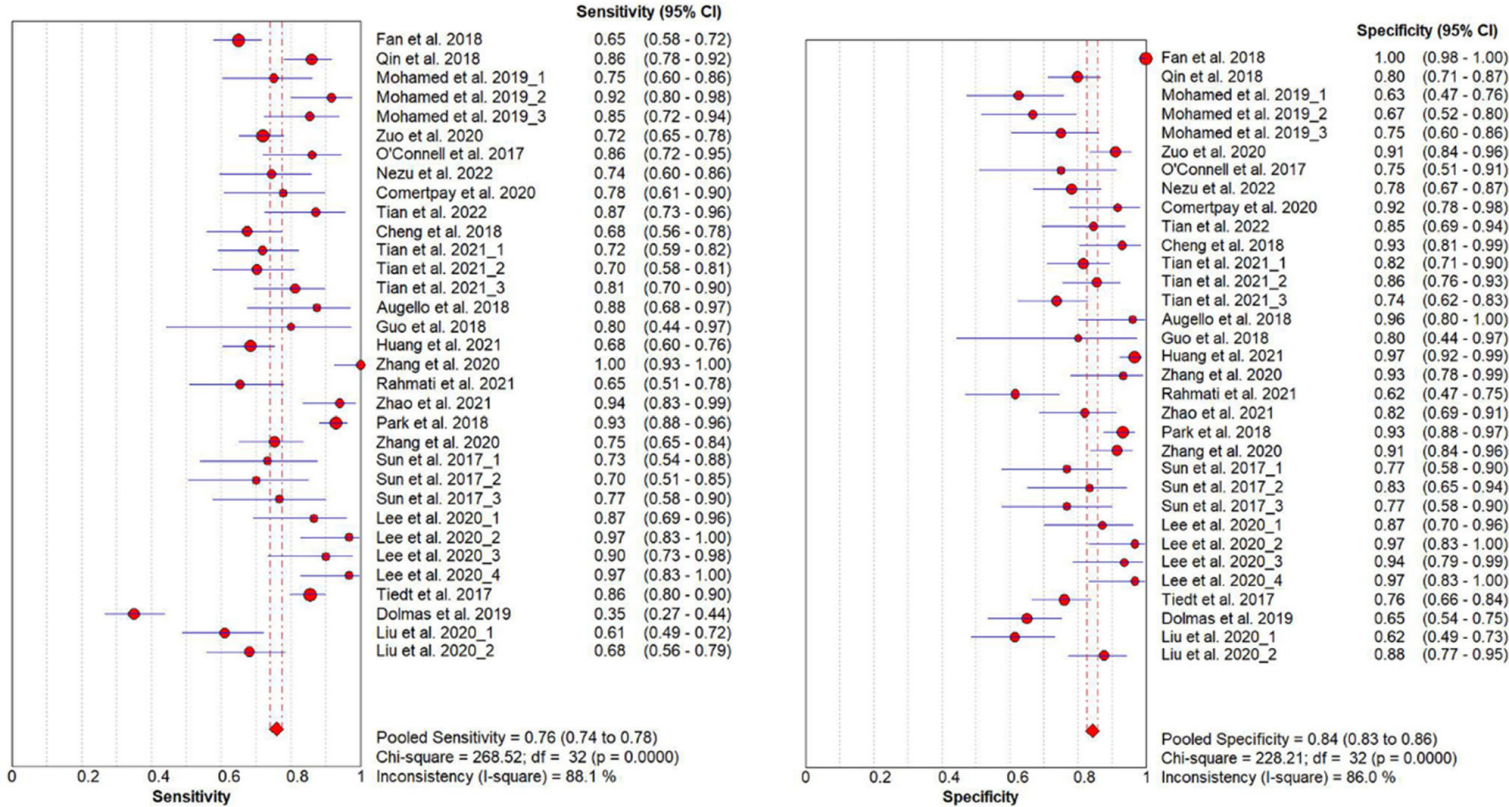
Forest plots of sensitivity and specificity of blood biomarkers for diagnosis of IS. The pooled sensitivity is 0.76 (0.74–0.78); the pooled specificity is 0.84 (0.83–0.86).

On account of the significant heterogeneity among all the studies analyzed (*I*^2 ^> 50%, 88.1% for sensitivity; 86.0% for specificity; *p* < 0.001 for both), the random-effect model was adopted for meta-analyses. As for the threshold effect, the overall Spearman's correlation coefficient was −0.27 with a *p* value of 0.13, indicating no evidence of a threshold effect. The pooled AUC of all studies for meta was 0.89, suggesting a moderate diagnostic value (Table 3, Fig. 3). The pooled sensitivity and specificity were 0.76 (0.74–0.78) and 0.84 (0.83–0.86), respectively (Table 3, Fig. 4). The pooled PLR was 4.91 (3.69–6.53), meaning that blood biomarkers had a 4.91 times possibility of accurately diagnosing IS (Table 3). A pooled NLR of 0.26 (0.20–0.32) suggested a 23% likelihood of mistaking people with IS for those without IS (Table 3). A pooled DOR of 23.14 (14.15–37.84) indicated good correctness of diagnosis (DOR > 1; Table 3).

### Subgroup analyses and meta-regression analysis

Subgroup and meta-regression analyses were conducted to further explore the potential source of heterogeneity, considering the indicated significant heterogeneity (*I*^2 ^> 50%; *p* < 0.001). The groups were stratified based on setting, small/large sample size, target IS population, single/multiple blood biomarkers, and ethnicity. The detailed diagnostic accuracy of subgroups is shown in Table 3. Firstly, the blood biomarkers from other settings [AUC 0.91, sensitivity 0.71 (0.66–0.75), specificity 0.83 (0.78–0.87), DOR 16.95 (2.41–119.25)] demonstrated better diagnostic performance than inpatient setting [AUC 0.88, sensitivity 0.76 (0.74–0.78), specificity 0.84 (0.83–0.86), DOR 21.66 (14.15–33.16)]. Secondly, the sample size could impact diagnostic efficacy of blood biomarkers for diagnosis of IS, with the small sample size [<100; AUC 0.92, sensitivity 0.86 (0.82–0.88), specificity 0.82 (0.79–0.86), DOR 30.87 (16.31–58.42)] higher than the large one [≥100; AUC 0.86, sensitivity 0.73 (0.71–0.75), specificity 0.85 (0.83–0.87), DOR 17.57 (8.84–34.91)].

**Figure 5.**
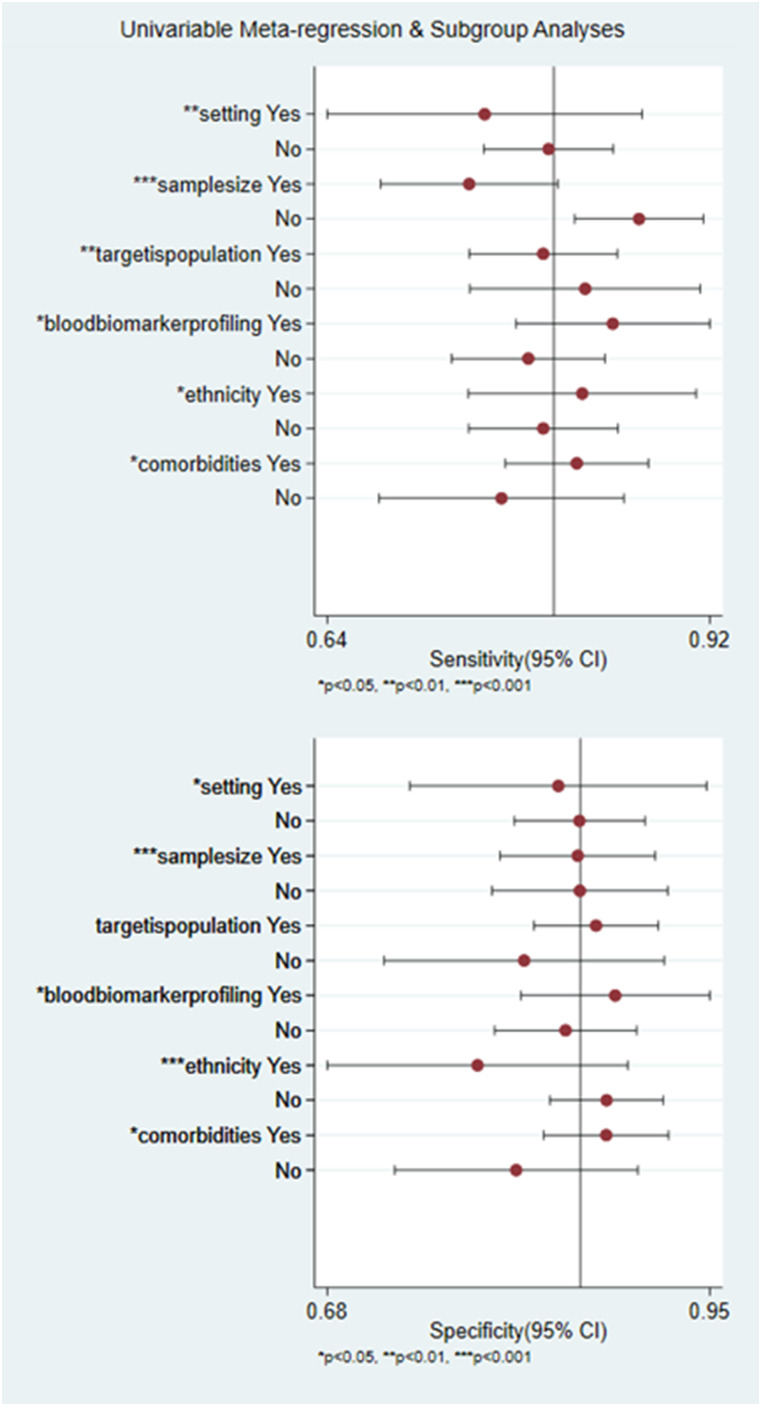
Univariable meta-regression and subgroup analyses for the heterogeneity.

Thirdly, blood biomarkers from AIS patients showed higher diagnostic value than IS and others [acute lacunar infarction, AIS with active colorectal cancer (CRCIS), cardioembolic stroke (CES), and cancer-associated IS (CAS); AUC 0.91 vs 0.87 and 0.84, sensitivity 0.77 (0.75–0.80) vs 0.82 (0.78–0.85) and 0.68 (0.64–0.72); specificity 0.88 (0.85–0.90) vs 0.80 (0.76–0.84) and 0.81 (0.78–0.84), DOR 32.72 (17.38–61.60) vs 17.74 (7.85–40.10) and 12.91 (3.69–45.16)]. In addition, the diagnostic performance of multiple blood biomarkers [individual blood biomarkers combined as a set; AUC 0.91, sensitivity 0.81 (0.78–0.83), specificity 0.86 (0.83–0.88), DOR 30.20 (19.44–46.91)] was superior to single biomarker [AUC 0.88, sensitivity 0.73 (0.71–0.75), specificity 0.84 (0.82–0.86), DOR 19.74 (10.56–36.89)]. Furthermore, the diagnostic value of blood biomarkers from the Caucasian was higher than the Asian and the African [AUC 0.90 vs 0.89 and 0.75, sensitivity 0.77 (0.73–0.80) vs 0.75 (0.73–0.77) and 0.84 (0.77–0.90), specificity 0.81 (0.77–0.85) vs 0.86 (0.85–0.88) and 0.68 (0.60–0.76), DOR 23.52 (2.93–188.62) vs 23.92 (14.81–38.64) and 11.76 (4.52–30.56)]. Blood biomarkers from those with comorbidities [AUC 0.92, sensitivity 0.77 (0.75–0.79), specificity 0.85 (0.83–0.87), DOR 31.55 (16.00–62.20)] showed a better diagnostic performance than those not reporting comorbidities [AUC 0.84, sensitivity 0.74 (0.70–0.77), specificity 0.82 (0.79–0.85), DOR 14.21 (7.71–26.19)]. As for meta-regression analysis, it is suggested that setting, sample size, target IS population, blood biomarker profiling, ethnicity, and comorbidities might all be the sources of heterogeneity (Fig. 5).

#### Publication bias

**Figure 6.**
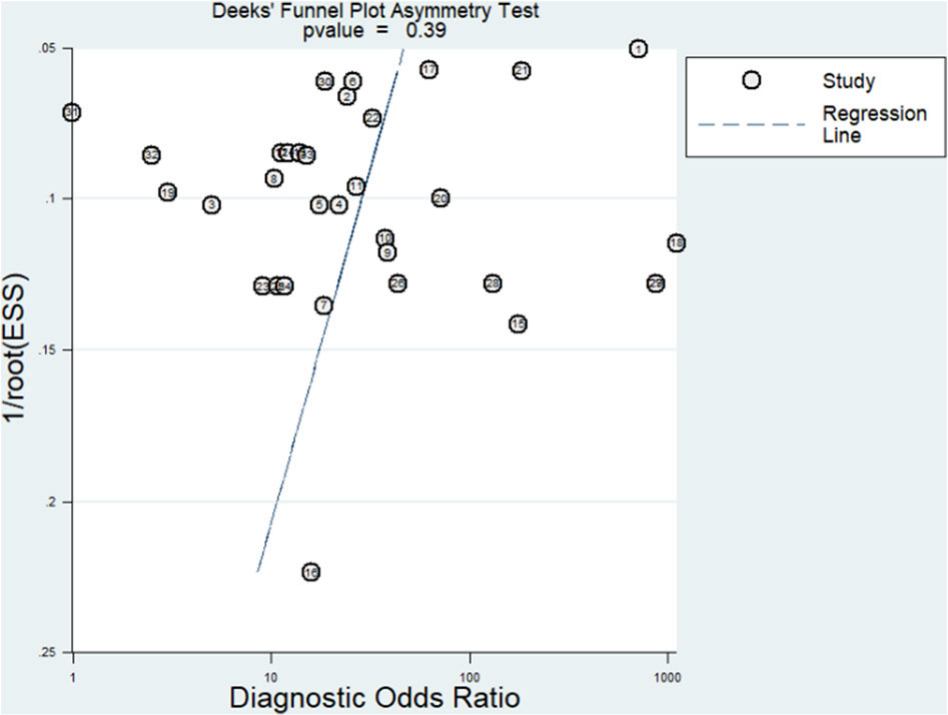
Deek's funnel plot to estimate publication bias.

Publication bias was evaluated by Deeks’ funnel plot asymmetry test, which revealed that there was no publication bias (*p* = 0.39; >0.10; Fig. 6).

#### Clinical utility of blood biomarkers for diagnosis of IS

**Figure 7.**
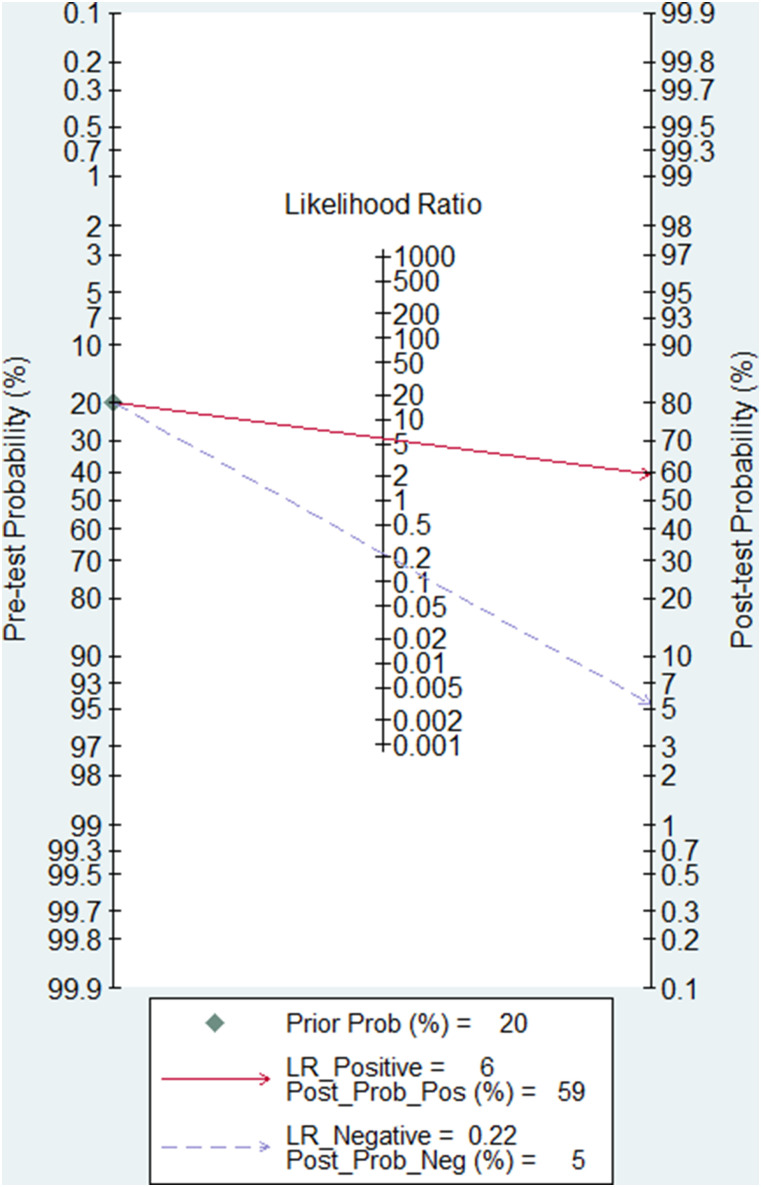
Fagan nomogram to evaluate the clinical utility of blood biomarkers for diagnosis of IS.

Fagan nomogram was applied to assess the clinical utility of blood biomarkers for the diagnosis of IS. When the pretest probability was set to 20%, the posttest probability of blood biomarkers increased to 59% of PLR and 5% of NLR (Fig. 7).

## Discussion

Stroke is the leading cause of death and disability globally and requires rapid diagnosis since “time is the brain” (Campbell and Khatri, 2020; Misra et al., 2020). Moreover, the immediate application of the therapy and medication to stroke patients also depends on the precise and fast diagnosis of stroke (Makris et al., 2018). Since the potential blood biomarkers in the blood test can be used to differentiate IS from stroke mimics and intracerebral hemorrhage (Misra et al., 2020), a rapid and simple blood test could be of diagnostic value and assist the clinical and imaging diagnosis of IS and risk stratification in confirmed cases (Whiteley et al., 2008). Blood biomarkers as an objective tool to measure molecular characteristics can help stroke diagnosis and management (Bustamante et al., 2017; Kamtchum-Tatuene and Jickling, 2019). Some systematic reviews have identified the ability of biomarkers in stroke management, including the study of Palà et al. and Priya et al. (Palà et al., 2020; Dev et al., 2022).

Our study primarily focused on evaluating the diagnostic accuracy of blood biomarkers specifically for IS. In contrast, the study by Misra et al. (2020) aimed to assess the diagnostic capability of blood biomarkers for differentiating IS from intracerebral hemorrhage, stroke mimics, or healthy controls (Misra et al., 2020). While Misra et al. (2020) explored these distinctions, our study concentrated solely on the accuracy of biomarkers for diagnosing IS, rather than differentiating between various stroke subtypes. Additionally, Misra et al. (2020) noted that previous research had not established the use of blood biomarkers to distinguish transient ischemic attacks (TIAs) from healthy individuals or stroke mimics. However, the study by Dolmans et al. (2019), which was included in our review, demonstrated that several biomarkers could differentiate transient IS from healthy controls. To the best of our knowledge, our study is the most recent effort focused exclusively on assessing the accuracy of blood biomarkers in diagnosing IS.

In this study, we included 29 articles for systematic analysis, with 23 applicable (3,494 participants) for meta-analysis to further explore the diagnostic value of blood biomarkers for IS. The results of our study suggested that blood biomarkers are promising biomarkers for the diagnosis of IS. Due to the heterogeneity detected, the random-effect model was applied. The pooled AUC of 0.89 indicated considerable precision of blood biomarkers in diagnosing IS, and the pooled sensitivity and specificity were >0.70. Additionally, DOR [23.14 (14.15–37.84)], amalgamating both sensitivity and specificity, also positively correlated with AUC, and the biased trial presentation reveals great discriminatory test performance (Glas et al., 2003). Ranging from 0 to infinity, the higher the DOR, the better the discriminatory ability.

The Fagan test showed that with a pretest probability of 20%, blood biomarkers had a 59% probability of correctly diagnosing IS in people with IS and a 6% probability of wrong diagnosing IS in people without IS. All these results demonstrated the high value of blood biomarkers in diagnosing IS, thus promoting the early diagnosis of IS in clinical practice. There was no threshold effect, as shown by Spearman's correlation (0.27; *p* = 0.13), suggesting that the threshold effect is not a source of heterogeneity. The result of the meta-regression indicated that the possible sources of heterogeneity among the included studies for meta-analysis might be setting, sample size, target IS population, blood biomarker profiling, and ethnicity. Furthermore, there was no publication bias, as suggested by Deeks’ funnel plot asymmetry test (*p* = 0.39). Our meta-analysis showed that blood biomarkers could be a potential method to help IS diagnosis, although blood biomarkers for IS diagnosis are not indicated in guidelines for stroke diagnosis. Our meta-analysis revealed a moderate diagnostic value of blood biomarkers. Since stroke diagnosis is challenging for healthcare providers, missed or delayed diagnosis of AIS has been reported in as high as 9% of confirmed stroke cases (Saleh Velez et al., 2021). We hope that our findings will provide a new perspective to be considered for IS diagnosis. We suggest that future research should study the combination of stroke signs and symptoms evaluation, imaging for stroke (CT and MRI), and blood biomarkers to increase diagnostic stroke accuracy, which would decrease poststroke morbidity and mortality.

Our meta-analysis results not only illustrated how the blood biomarkers explicit the significant accuracy for stroke diagnosis but also revealed one concerning point about the sample size that could impact the efficacy of blood biomarkers for IS diagnosis. According to previous literature (Al-Mekhlafi et al., 2020), a too-small sample size could diminish the ability to validate a significant effect, resulting in the insufficient power of the study to capture the actual difference between groups. However, when looking back at our results, it shows that the studies that contained a small sample size (<100) had a higher AUC, sensitivity, specificity, and DOR than the large ones. This concern could be explained by considering the nature and limitations of biomarker studies. To explain, the complexity of human responses is often too complex to be based on one biomarker and needs advanced technology to identify the candidate biomarkers (Al-Mekhlafi et al., 2020). On the other hand, using a large sample size might not be feasible due to costly methods and ethical concerns (Al-Mekhlafi et al., 2020). Therefore, in the included studies containing <100 samples, advanced technology such as microarray, metabolic profiling, DNA, RNA, and protein extraction were used, resulting in a vast number of biomarker candidates and combinations of biomarkers with higher accuracy. Meanwhile, in the larger sample-sized studies, real-time polymerase chain reaction and other inflammatory markers were often utilized, which may have less efficacy in detecting IS.

To our knowledge, no literature has compared the individual diagnostic value of blood biomarkers in AIS and IS. Our meta-analysis showed that blood biomarkers from AIS patients showed higher diagnostic values than IS. However, previous studies did investigate other kinds of parameters for diagnosing IS. For example, a history of previous stroke or TIA and small vessel strokes were mentioned as having a significant relation with recurrent IS rate with pooled RR 2.5 (95% CI 2.1–3.1) and 0.3 (95% CI 0.1–0.7), respectively (Kauw et al., 2018). Furthermore, multiple lesions with multiple stages of brain infarction shown on MRI had increased recurrent IS occurrence with pooled RR 1.7 (95% CI 1.5–2.0; Kauw et al., 2018).

Our study discovered that the diagnostic performance of multiple blood biomarkers was superior to a single biomarker. This finding yielded a similar result to a previous study aimed to estimate the value of combined multipanel diagnostic accuracy of troponin-I, N-terminal proatrial natriuretic peptide, cystatin-C, and high-sensitivity C–reactive protein (hs-CRP) in patients with coronary artery diseases (CAD) at the time of admission, suggesting that combined assessments of two biomarkers for diagnostic performance for CAD were better than single biomarkers (Al-Mumin et al., 2020). Nevertheless, one study on IS patients suggested that no ideal blood marker exists for IS diagnosis (An et al., 2013). Utilizing multiple blood markers, the researcher failed to discover a significant marker panel that improves clinical IS diagnosis. Although some blood proteins, including interleukin-6 (IL-6), S100B, and MMP-9, are significantly elevated in the acute phase of IS, they did not enhance the diagnostic value of clinical assessment tools (An et al., 2013).

However, it is important to note that many of the biomarkers evaluated, while showing improved diagnostic performance when combined, are also involved in other cardiovascular and metabolic diseases (Omran et al., 2022; Thupakula et al., 2022). This lack of specificity means that while combining multiple biomarkers may improve diagnostic capacity, it does not completely resolve the issue of distinguishing IS from other conditions. The biomarkers used in our study, such as IL-6 and MMP-9, are not exclusively specific to IS and are also expressed in other diseases, which can lead to challenges in accurate diagnosis and potential FPs. As a result, while the combination of biomarkers may offer better diagnostic performance compared with single biomarkers, it still requires further refinement to enhance specificity.

Therefore, healthcare professionals should await further investigations of blood biomarkers before using them in regular clinical practice for IS diagnosis. The combination of multiple blood markers seems to improve the capacity to diagnose IS, but additional work is necessary to identify a model of combined blood biomarkers with higher specificity. Further research is needed to validate these findings and develop more specific biomarker panels to improve the accuracy of IS diagnosis.

We found heterogeneity in different ethnic groups, as suggested by meta-regression analysis and subgroup analyses. Compared with African individuals with IS, blood biomarkers from Caucasians and Asians showed higher accuracy in diagnosing IS. One possible reason could be that only one African study was included with a small sample size, which could not sufficiently represent the status of a large range of patients. Some studies also revealed that ethnicity contributed to between-study heterogeneity. For example, in a systematic review of the diagnostic performance of ischemia-modified albumin in stroke, it was found that the diagnostic performance differed between studies based on the continent of Europe or Asia (Shi et al., 2021). This finding is consistent with a previous meta-analysis of dietary linoleic acid and stroke risk, which showed statistical differences between whites and Asians (W. Zhang et al., 2020).

### Strengths and limitations

Our results demonstrate that blood biomarkers have promising clinical utility in diagnosing IS. Furthermore, the heterogeneity in this study has improved the generalizability of the blood biomarkers as a diagnostic tool for IS. Therefore, our result could help to draw new assumptions, develop future research designs, and facilitate transparent decisions about the blood biomarkers for IS diagnosis. Moreover, this meta-analysis has shown improved statistical power in identifying the biomarkers for IS. The increased statistical power is evident based on the pooled AUC, sensitivity, specificity, PLR, NLR, and DOR falling in the optimal values. The quality appraisal of included studies was addressed using QUADAS-2, and the publication bias was evaluated by Deeks’ funnel plot asymmetry test, which revealed no publication bias.

Some limitations of this study were noted. First, 19 of the 29 included studies are from China; a more even distribution of nationalities and ethnicities of stroke patients would be ideal for applying findings to the global population. Second, many included studies had relatively small sample sizes, which might hinder the diagnostic efficacy. However, the inclusion of many studies may help to alleviate this risk. Moreover, the participant selection process in many included studies remains relatively unclear, which raises the risk of bias, although we did not find publication bias in our study. Additionally, the biomarkers assessed in our study are not specific to IS alone and may also be expressed in other cardiovascular and metabolic diseases, potentially affecting diagnostic accuracy and leading to challenges in distinguishing IS from other conditions. Finally, the varied comorbidity data precluded further investigation on the impact of certain comorbidity on the diagnostic performance of biomarkers and thus may impact the generalizability of our findings to specific subpopulations with comorbid conditions. Future research could aim to explore the influence of certain comorbidities on biomarkers’ diagnostic value. All limitations noted could be addressed by future clinical trials in large-scale populations and long-term assessments.

### The implication to future research and conclusion

Our meta-analysis concluded that blood biomarkers have sufficient diagnostic accuracy for IS diagnosis and have great potential to be used in routine clinical practice. Using combined biomarkers rather than a single biomarker appeared to be more effective in diagnosing IS, as it could improve accuracy. The quality and diagnostic accuracy of the included studies seemed to be fair. Additionally, there was no evidence of a threshold effect in the diagnostic performance of blood biomarkers and no publication bias among the included studies. Therefore, the existing literature is sufficient to be used as a foundation for future clinical practice guidelines. As IS biomarkers can potentially enhance the outcome of IS survival, more research is required to examine the burden and cost of testing to form a complete picture of the harms and benefits and improve the reporting of future studies.

## Synthesis

Reviewing Editor: Selva Baltan, Oregon Health &Science University Department of Surgery Decisions are customarily a result of the Reviewing Editor and the peer reviewers coming together and discussing their recommendations until a consensus is reached. When revisions are invited, a fact-based synthesis statement explaining their decision and outlining what is needed to prepare a revision will be listed below. The following reviewer(s) agreed to reveal their identity: Ami Raval.

Synthesis Statement for Author (Required):

Satisfactory revisions are received
